# Factors Associated with Knowledge of and Willingness for Adult Male Circumcision in Changsha, China

**DOI:** 10.1371/journal.pone.0148832

**Published:** 2016-02-09

**Authors:** Mingqiang Zeng, Ling Wang, Caifang Chen, Fanchang Zeng, Liang Huang, Ruizhi Xue, Junjie Chen, Benmin Gao, Zhengyan Tang

**Affiliations:** 1 Department of Urology, Xiangya Hospital, Central South University, Changsha, Hunan, China; 2 College of Public Health, Ohio State University, Columbus, Ohio, United States of America; 3 Department of Geriatric Medicine, Xiangya Hospital, Central South University, Changsha, Hunan, China; Rega Institute for Medical Research, BELGIUM

## Abstract

**Background:**

Male circumcision (MC) has been shown to reduce the risk of male genital diseases. MC is not commonly practiced among Chinese males and little is known about the factors associated with their knowledge of and willingness for MC. This study was to explore the knowledge regarding the foreskin among Chinese males and to identify factors associated with their willingness to undergo circumcision.

**Methods:**

A total of 237 patients with redundant prepuce/phimosis were interviewed through face-to-face interviews. The items on the questionnaire included: demographics, an objective scale assessing knowledge about the foreskin, willingness to have MC, the attitudes of sexual partners and doctors toward redundant prepuce/phimosis, and the approaches that patients used to acquire knowledge regarding the prepuce. Univariate analysis and multiple logistic regression analysis were performed to identify factors that are associated with willingness to be circumcised (WTC).

**Results:**

A total of 212 patients completed the interview. Multivariable logistic regression showed that three factors were significantly associated with WTC: being married (OR = 0.43), perceiving redundant prepuce/phimosis as a disease (OR = 1.93), and if a patient’s partner supported MC (OR = 1.39). 58% (n = 122) had received information about the foreskin from another party: 18% (n = 37) from school, 8% (n = 17) from family, 17% (n = 36) from friends, 27% (n = 57) from health care providers. About 4% (n = 8) believed that their partners disliked their redundant prepuce/phimosis. 20% (n = 42) had received doctors’ advice to undergo circumcision.

**Conclusion:**

Knowledge about the foreskin was low among Chinese males. Our study elucidates the factors associated with WTC and suggests that more education of the population about the foreskin can help improve the recognition of a correctible abnormality and help patients assess the potential role of MC in their health.

## Introduction

Redundant prepuce/phimosis is common in men. Male circumcision is the earliest human urologic surgery performed in many countries [1,2]. The effectiveness of male circumcision (MC) in preventing transmission and decreasing the risk of urologic disease during the neonatal period [3,4] and sexually transmitted infections (STIs) over the lifetime [5,6] has been reported previously. Studies suggest that uncircumcised men have higher risk of acquiring STIs including syphilis, gonorrhoea, and chlamydia than circumcised men [7,8]. Randomized controlled trials (RCTs) in Africa have shown that MC reduced the risk of HIV infection by 50% to 60% in heterosexual men [9,10,11], as well as the risk of herpes simplex virus type 2 (HSV-2) infection and human papillomavirus (HPV) infection [12]. Among the female partners of circumcised men, bacterial vaginosis was reduced by 40% and trichomonas vaginalis infection was reduced by 48% [13]. It appears that in the settings studied, MC reduces the risk of STIs in men (particularly viral) as well as STI transmission to their female partners [14]. Additional benefits of male circumcision may include a lower risk of penile cancer [15], a lower risk of foreskin infections, and easier genital hygiene. Meanwhile, a systematic review suggested that male circumcision has no adverse effect on sexual function, sensitivity, sexual sensation, or satisfaction [16]. MC is recommended by the Joint United Nations Program on HIV/AIDS (UNAIDS) and is recognized as an additional and important strategy to prevent heterosexually acquired HIV infection in men [17]. However, MC is not commonly practiced among Chinese males. The prevalence of MC worldwide is almost 30%; only 5% of Chinese males have been circumcised [18].

While the knowledge of circumcision or the acceptability of MC have been studied in men and women in sub-Saharan Africa [19,20], the United States [21], Thailand [22], and Western China [23], little is known about the factors related to the knowledge of foreskin and willingness to have MC. Partners’ and doctors' attitudes regarding circumcision and the manner by which men acquire knowledge about the prepuce have not been reported in the literature. To begin to examine this issue, we studied a group of Chinese men who have abnormal foreskin in the form of redundant prepuce/phimosis and to identify the factors associated with MC in Changsha, China.

## Methods

### Study design and subjects

This study was conducted at the Outpatient Department of Urology, Xiangya Hospital, Central South University in Changsha, China. Between September 2014 and March 2015, 744 male patients had physical examinations in the outpatient department, among whom we recruited 237 patients who had redundant prepuce/phimosis, aged 18 to 60 years. Patients with hearing or speech impairment or who had undergone MC were excluded. Face-to-face interviews were conducted with the study population to collect data. This study was approved by the Medical Ethics Committee of the Xiangya Hospital.

### Questionnaires and data management

A 14-item validated questionnaire was adapted from other studies with the primary aim to collect data on foreskin knowledge and willingness to be circumcised as an effective strategy to prevent genital diseases [23]. The questionnaire has five sections, including patient demographics, general knowledge about redundant prepuce/phimosis, willingness to accept MC, the female partner or doctors’ awareness of relevant disease, and the manner by which men obtained knowledge about the foreskin. Primary outcome variables were assessed by asking close-ended questions, such as ‘‘Do you know that redundant prepuce/phimosis is a disease?” with response categories of ‘‘Yes/No.”

To assess knowledge about redundant prepuce/phimosis, seven questions were asked to collect information on general knowledge about the prepuce, such as whether they know that redundant prepuce/phimosis could affect sexual intercourse. For foreskin knowledge, each answer was given a score of 1 if the answer was ‘Yes’, and a score of 0 if the answer was ‘No’. Willingness to accept circumcision was assessed with the question “are you willing to be circumcised?”, and the response categories were ‘‘Yes/No”. We also asked four questions about the attitudes of sexual partners and doctors towards the disease, and the response categories were ‘‘Yes”, “No” and “Don’t care/ Never checked genitals”. The last two questions were about foreskin knowledge and how patients obtained it: “Do you have some knowledge about foreskin?” and “How did you learn it?”

All data were collected by trained research assistants (RAs). After the subjects provided their written informed consent to participate in the study, RAs conducted the detailed interviews following the structured guidelines.

### Analysis

Descriptive statistics were developed for each of the variables corresponding to specific questions in the survey, including demographics, knowledge of redundant prepuce/phimosis and the family or doctors’ awareness to the disease. Chi-square tests were performed to compare patients’ WTC by demographics (e.g., age and ethnicity), education, knowledge about the effects of redundant prepuce/phimosis on intercourse satisfaction, and the attitude of patients’ partners toward MC. One-way ANOVA was performed to analyze foreskin knowledge score by demography and other characteristics such as knowledge about the effects of redundant prepuce/phimosis on intercourse satisfaction and the attitude of patients’ partners toward MC. All statistical tests were two-sided with a significance level of p < 0.05. Multivariable logistic regression analysis was performed to identify factors associated with the WTC. Variables that showed a statistically significant association (p <0.05) with WTC were included in multivariate analyses. All the data were analyzed using SPSS for windows Version 18.0 (SPSS, Chicago, IL).

## Results

### Demographic characteristics

A total of 212 patients completed the interview (completion rate: 89.5%); the average age of participants was 37.5. 95% of participants were Han ethnicity, 79% were married, 78% of them had at least one child, 77% had at least high school education, and 50% of them were born in the city of Changsha (Table 1). Over 90% of respondents had sexual intercourse in the past year (Table 1).

**Table 1:** Demographic characteristics of the study population.

Variables	n	Percentage (%)
Total	212	100
Age		
18–30	67	32
31–45	94	44
46–60	51	24
Ethnic group		
Han	201	95
Other minorities	11	5
Occupation		
Industrial	58	27
Government-affiliated institutions	88	42
Business service	44	21
Student	22	10
Education level		
Junior high school or below	49	23
High school or above	163	77
Region of birth		
Rural	109	51
Urban	103	49
Marital status		
Married	168	79
Unmarried	44	21
Fertility status		
At least one child	166	78
No child	46	22
Had sexual intercourse in the past year		
Yes	198	93
No	14	7

### Knowledge about the prepuce

Overall, knowledge regarding redundant prepuce/phimosis was low. 41% thought that redundant prepuce/phimosis is a disease; 55% (n = 116) recognized that their foreskin was too long or phimotic; 38% (n = 81) knew that redundant prepuce/phimosis may cause balanoposthitis and cancer; 51% (n = 108) believed that phimosis can affect sexual intercourse; 43% (n = 92) knew that redundant prepuce/phimosis is associated with sexual partners’ gynecological inflammation and cervical cancer; and 44% (n = 93) thought that phimosis could affect penis growth in children. 92% (n = 195) of the respondents reported cleaning smegma frequently (Table 2).

**Table 2:** Knowledge of Chinese men regarding redundant prepuce /phimosis.

Variables	n	Yes (%)
1. Do you know that redundant prepuce/phimosis is a disease?	86	41
2. Do you feel that your foreskin is too long or phimotic?	116	55
3. Do you clean smegma frequently?	195	92
4. Do you know that redundant prepuce/phimosis may cause balanoposthitis and cancer?	81	38
5. Do you know that phimosis can affect sexual intercourse?	108	51
6. Do you know that redundant prepuce/phimosis will increase the morbidity of sexual partners’ gynecological inflammation and is associated with cervical cancer?	92	43
7. Do you know that phimosis will affect penis growth in children?	93	44

### Means by which patients gained information about the prepuce

4% thought that their partner did not like their redundant prepuce/phimosis and 8% of respondents’ partner had suggested the respondent undergo circumcision. 30% of the respondents reported that during a previous physical examination they had been informed by a doctor about their redundant prepuce/phimosis, and 20% of them received a doctor’s advice to be circumcised (Table 3). 58% of patients had received some knowledge about the foreskin from an outside source: 18% of them obtained the knowledge from school, 8% from family, 16% from friends, and 27% from health care providers (Table 3).

**Table 3:** The attitude of patients’ partner and doctor toward redundant prepuce/phimosis and the approaches that participants used to obtain foreskin knowledge.

Variables	n	Percent
1. Does your partner dislike that you have redundant prepuce/phimosis?		
Yes	8	4
No	96	45
Do not care	108	51
2. Has your wife/girlfriend suggested that you have a circumcision?		
Yes	16	8
No	71	34
Do not care	125	59
3. During any physical examinations in the past, has a doctor told you that you have redundant prepuce/phimosis?		
Yes	64	30
No	94	44
Never checked genitals	54	26
4. During any visit to a doctor or health care providers in the past, were you advised that you should be circumcised?		
Yes	42	20
No	116	55
Never checked genitals	54	26
5. Have you received any information, from any source, about the foreskin?		
Yes	122	58
No	90	42
6. How did you obtain the information about the foreskin? (Check all that apply)		
School	37	18
Family	17	8
Friends	36	16
Health care providers	57	27

### Knowledge of foreskin

Table 2 presents responses to questions about the foreskin, and Table 4 presents the averages of the data, based on seven items scored on a Yes/No scale. The average foreskin knowledge score was 3.6 ± 1.9. By ANOVA, the score significantly differed by age, occupation, education, and birthplace (P<0.05): younger men with high school or more education, born in urban area and being a student had significantly higher scores than other groups. No significant variation was observed in the score by marital status. Compared to those who had at least one child, those without child had significantly higher foreskin knowledge scores.

**Table 4:** Foreskin knowledge score by age, occupation, education level, birthplace, marital status and fertility status.

Variables	Foreskin knowledge score (mean ± SD)	P value
Age		
18–30	3.9 ± 1.7	0.03
31–45	3.7 ± 1.8	
46–60	3.1 ± 1.8	
Occupation		
Industrial	3.7 ± 1.5	0.03
Government-affiliated institutions	3.8 ± 1.9	
Business service	3± 1.6	
Student	4.2 ± 1.5	
Education level		
Junior high school or below	3.2 ± 1.7	0.03
High school or above	3.8 ± 1.8	
Region of birth		
Rural	3.3 ± 1.9	0.01
Urban	4± 1.6	
Marital status		
Married	3.7 ± 1.9	0.54
Unmarried	3.5 ± 1.9	
Fertility status		
At least one child	3.5 ± 1.7	0.01
No child	4.2 ± 1.7	

### Factors associated with the willingness to be circumcised

All the factors excepting occupation and urban/rural residence were significantly associated with the willingness to be circumcised (Table 5). The multivariable logistic regression analysis identified three factors that were associated with WTC (Table 6), including: being married (OR, 0.43; 95%CI, 0.20–0.95), believing redundant prepuce/phimosis to be a disease (OR, 1.93; 95%CI, 1.06–3.52), and wife/girlfriend’s negative attitude toward his redundant prepuce/phimosis (OR, 1.39; 95%CI, 1.03–1.87).

**Table 5:** Factors Associated with WTC.

Variables	WTC		χ^2^	p value
	Yes n (%)	No n (%)		
Total	133(100)	79(100)		
Age				
18–30	50(38)	17(22)	7.2	0.03
30–45	57(43)	37(47)		
45–60	26(20)	25(32)		
Occupation				
Industrial	32(24)	26(33)	3.3	0.35
Government-affiliated institutions	61(46)	27(34)		
Business service	26(20)	18(23)		
Student	14(11)	8(10)		
Education level				
Junior high school or below	49(37)	43(54)	6.2	0.01
High school or above	84(63)	36(46)		
Region of birth				
Urban	64(48)	39(49)	0.03	0.86
Rural	69(52)	40(51)		
Marital status				
Married	99(74)	69(87)	5.02	0.03
Unmarried	34(26)	10(13)		
Fertility status				
At least one child	98(74)	68(86)	4.5	0.03
No child	35(26)	11(14)		
Do you know that redundant prepuce/ phimosis can affect sexual life?				
Yes	75(56)	33(42)	4.2	0.04
No	58(44)	46(58)		
Have your wife/girlfriend suggested you to accept circumcision?				
Yes	15(11)	1(1)	7.1	0.01
No	118(89)	78(99)		

**Table 6:** Multivariable analysis of WTC.

Variables	Adjusted OR (95% CI)	P value
Marital status		
Unmarried	1	
Married	0.43(0.20–0.95)	0.04
Do you know that redundant prepuce/phimosis is a disease?		
No	1	
Yes	1.93(1.06–3.52)	0.04
Does your partner dislike that you have redundant prepuce/phimosis?		
No	1	
Yes	1.39(1.03–1.87)	0.03

OR: odds ratio; CI: confidence interval.

Adjusted for age, marital status, fertility status, region of birth, knowledge of Chinese men regarding redundant prepuce/phimosis (including items 1–2, 4–6 in Table 2) and the attitude of patients’ partner and doctor toward redundant prepuce/phimosis (including items 1–4 in Table 3).

## Discussion

This is the first study that directly investigated patient knowledge and views of and indirectly investigated partners’ and doctors’ awareness of and attitude toward redundant prepuce/phimosis among Chinese men, as well as the approaches used for men to acquire knowledge about foreskin. The findings indicate that partners revealed little concern about abnormality of their male partners’ foreskin. Also noted was that few males received genital examination and professional advice for redundant prepuce/phomisis. In addition, nearly half of respondents have never received any information about the foreskin from the common external information sources. Speculatively, these factors may be associated with low MC among Chinese. Thus a broad education campaign that includes men, partners, doctors, nurses and in fact the entire population is much needed if broader male circumcision is a goal.

Our findings showed that foreskin knowledge score was low, with an average of 3.6 ± 1.9 (out of 7). The score is consistent with the low MC knowledge index in rural Zimbabwe[24]. Only 41% believed that redundant prepuce/phimosis is abnormal. 55% believed that they have redundant prepuce/phimosis, a higher percentage than that reported by Yang in Western China (22%) [23]. Knowledge that redundant prepuce/phimosis affects sexual intercourse and their partner’s reproductive health was present in 51% and 43% of our respondents respectively. In the present study, about 92% of respondents clean smegma frequently.

We found that young men below 30 years were more willing to be circumcised than men over 45 years. Our findings are consistent with the findings in the Dominican Republic and Kenya [25,26]. Our results showed that more young males knew that MC can prevent penile inflammation and cancer. The higher willingness for MC among younger males may result from this fact. Moreover, younger men also paid more attention to their sexual hygiene and sexual health. Foreskin knowledge score was higher in students than in business service personnel. High school or further education was associated with a higher score as well.

Westercamp and Bailey reviewed 13 studies to identify factors associated with MC acceptability, including beliefs that MC leads to improved hygiene, protection from STIs and HIV infection, as well improved sexual pleasure and performance, and considered ethnicity, pain, culture and religion, cost, possible adverse events (AEs), and the potential for risk compensation (i.e., an increase in risky sexual behavior following MC) [21]. However, we identified several new factors that related to WTC, including education level, age, occupation, birthplace, marital status, fertility status, and sexual partner’s advice.

Interestingly, we found that unmarried men were more willing to be circumcised than married men although their foreskin knowledge scores were not significantly different. Significantly lower score or WTC were observed among those had at least one child compared to those without children. As could be reasonably expected, in this study population, patients without children tended to be younger. The younger population may tend to pay more attention to their personal hygiene and be more open to enhancing their foreskin knowledge and this may result in a willingness to be circumcised. Although the foreskin knowledge score among those born in rural areas was significantly lower than those born in urban areas (p = 0.01), the WTC was not significantly different.

Our findings showed that partners' attitudes played an important role in WTC. Patients whose partners cared about their redundant prepuce/phimosis were more likely to have WTC. This finding is consistent with previous studies. Herman-Roloff et al. [20] reported that increasing involvement of women’s groups to mobilize their partner to accept MC is one of the effective approaches to promote MC.

Our study has several limitations. First, the study group was selected from a population who had presented to urology clinic and had a high proportion of patients with abnormal foreskin anatomy. In addition, the attitude of sex partners and doctors about the disease were reported secondarily by the patients, which may result in recall bias and other biases. The subjects could have recognized the pattern of positive answers in the knowledge score questionnaire, and social desirability may have affected patients’ responses, both of which conceivably inflated the knowledge scores.

## Conclusions

This study showed that males in Changsha, China have little knowledge about foreskins. We identified factors that can be used for future programs to promote MC. Appropriate education and enhancing the attention of sexual partners and doctors to redundant foreskin/phimosis could greatly improve the willingness to undertake MC, suggesting that wider public programs are necessary to promote MC for reproductive health in China.
